# The impact of maternal depression during pregnancy on the risk of gestational diabetes mellitus: a meta-analysis

**DOI:** 10.3389/fendo.2025.1672527

**Published:** 2025-10-06

**Authors:** Nan Du, Limin Dai, Min Xu, Tingting Guan

**Affiliations:** ^1^ The Department of Endocrinology, Jiangsu University Affiliated People’s Hospital, Zhenjiang, Jiangsu, China; ^2^ Jiangsu Provincial Xuzhou Pharmaceutical Vocational College, Xuzhou, Jiangsu, China

**Keywords:** maternal depression, pregnancy, gestational diabetes mellitus, meta-analysis, mental health

## Abstract

**Background:**

Antenatal depression, defined as clinically significant depressive symptoms occurring during pregnancy, has been suggested to increase the risk of gestational diabetes mellitus (GDM), a glucose intolerance disorder with onset or first recognition during pregnancy. However, evidence regarding its relationship with GDM remains inconsistent. This meta-analysis aimed to quantitatively assess the association between antenatal depression and the risk of GDM.

**Methods:**

We systematically searched PubMed, Embase, Wanfang, and the Cochrane Library from inception to June 12, 2025, for observational studies reporting the association between depression during pregnancy and GDM. Pooled odds ratios (ORs) with 95% confidence intervals (CIs) were calculated using a random-effects model.

**Results:**

A total of eight studies were included in the meta-analysis. The pooled analysis showed that maternal depression during pregnancy was significantly associated with an increased risk of GDM (OR = 1.37, 95% CI: 1.20-1.54). Subgroup analyses based on country, depression assessment tool, and study design showed consistent results. Sensitivity analyses confirmed the stability of the results. No significant publication bias was detected.

**Conclusion:**

This meta-analysis suggests that maternal depression during pregnancy is associated with a significantly increased risk of developing GDM. Screening for depression in early pregnancy may represent a potential strategy to reduce the risk of GDM and improve maternal health outcomes.

## Introduction

Gestational diabetes mellitus (GDM) is one of the most common metabolic complications of pregnancy, affecting approximately 6.1%-15.2% of pregnancies worldwide (1). GDM is associated with increased risks of adverse maternal and fetal outcomes, including preeclampsia, macrosomia, cesarean delivery, and a heightened lifetime risk of developing type 2 diabetes mellitus (2). Understanding modifiable risk factors for GDM is essential for early intervention and prevention.

Maternal mental health, particularly antenatal depression, has gained increasing attention in recent years (3, 4). Affecting up to 20% of pregnant women worldwide, antenatal depression is associated with poor prenatal care, substance use, and adverse neonatal outcomes including preterm birth and low birth weight (5, 6). Emerging evidence further suggests its potential link to impaired glucose metabolism, possibly mediated by hypothalamic–pituitary–adrenal (HPA) axis activation, elevated cortisol, inflammation, and unhealthy lifestyle behaviors (7–10). In addition, the genome-wide association study, which combined the metabolomics and genetics data of pregnant women, found that the rs1260326 mutation of the glucose kinase regulatory protein (GCKR) gene was significantly associated with insulin sensitivity in pregnant women of multiple ethnicities (11). This indicates that genetic and metabolic factors are the basis for glucose regulation during pregnancy.

Despite growing interest in the interplay between mental health and metabolic disorders during pregnancy, findings on the relationship between antenatal depression and GDM risk remain inconsistent (12–14). Some studies have reported a positive association, while others found no significant link after adjusting for confounders such as age or BMI (12–18). Previous meta-analyses was constrained by small samples, and the absence of subgroup or sensitivity analyses, limiting the robustness of their conclusions (19). Therefore, we conducted a meta-analysis to quantitatively synthesize available evidence on the association between maternal depression during pregnancy and the risk of GDM. This study aims to clarify whether prenatal depression is an independent risk factor for GDM. The findings may provide important implications for integrated prenatal care addressing both psychological and metabolic health.

## Materials and methods

### Search strategy

A comprehensive literature search was conducted to identify studies examining the association between maternal depression during pregnancy and the risk of GDM. The databases PubMed, Embase, Cochrane Library, and Wanfang databases were systematically searched from inception to June 12, 2025. The search terms included combinations of keywords and MeSH terms related to “antenatal depression,” “maternal depression,” “gestational diabetes mellitus,” and “GDM”. No language or geographic restrictions were applied. In addition, the reference lists of included articles and relevant reviews were manually screened to identify additional eligible studies.

### Inclusion and exclusion criteria

Studies were included if they met the following criteria. Study design: cohort, case–control, or cross-sectional studies. Population: pregnant women. Exposure: depression diagnosed during pregnancy. Comparison: pregnant women without depression. Outcome: GDM incidence. Studies were excluded if they (1) included women with preexisting diabetes or depression; (2) lacked a comparison group; (3) are reviews, case reports, conference abstracts, or animal studies.

### Data extraction and quality assessment

Two independent reviewers screened the titles and abstracts, assessed full-text articles for eligibility, and extracted relevant data using a standardized data extraction form. Extracted information included first author, year of publication, country, study design, age, sample size, diagnostic criteria for depression and GDM, adjusted confounders, and effect estimates (OR) with 95% CI. Discrepancies were resolved by discussion or by a third reviewer. The quality of included studies was assessed using the Newcastle–Ottawa Scale (NOS). Studies scoring ≥6 on NOS was classified as high-quality study (20).

### Statistical analysis

Pooled odds ratios (ORs) and corresponding 95% confidence intervals (CIs) were calculated to estimate the association between maternal depression during pregnancy and the risk of GDM based on the random-effects model. Heterogeneity across studies was assessed using Cochran’s Q test (p < 0.05 considered significant) and the I² statistic (with I² > 50% indicating substantial heterogeneity). Sensitivity analyses were performed by sequentially removing each study to evaluate the stability of the pooled estimates. We have also conducted subgroup analyses based on the information available, such as countries, depression scale, or study design. Publication bias was assessed through visual inspection of funnel plots and statistically tested using Egger’s regression test and Begg’s rank correlation test. All statistical analyses were conducted using STATA software, version 12.0 (StataCorp, College Station, TX, USA), with a two-tailed p-value < 0.05 considered statistically significant.

## Result

### Study selection and study characteristics

**Figure 1:**
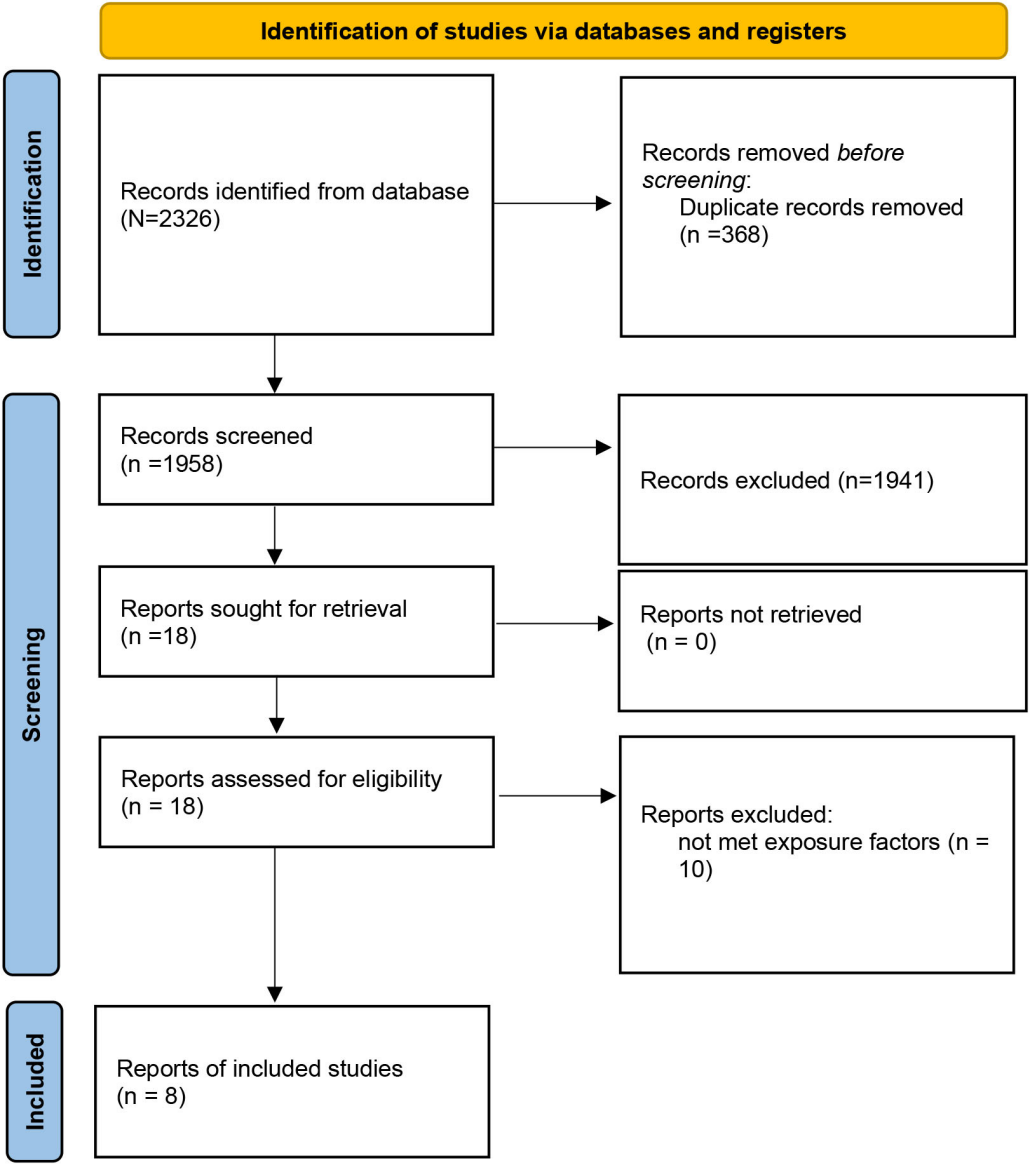
A flow diagram demonstrating the study selection process.

A total of 2,326 records were identified through database searching. After removing duplicates (n = 368), 1,958 articles remained for title and abstract screening. Based on inclusion and exclusion criteria, 18 full-text articles were assessed for eligibility, and finally, 8 studies (12–18, 21) were included in the meta-analysis (**Figure 1**). The included studies were published between 2013 and 2021, comprising a total of 125,451pregnant women. Study designs included five prospective cohort studies, one retrospective cohort study, and one case–control study. Depression was assessed using standardized tools such as the Edinburgh Postnatal Depression Scale (EPDS), Self-Rating Depression Scale (SDS), hospital anxiety and depression (HAD), International Classification of Diseases (ICD), or Center for Epidemiologic Studies Depression Scale-10 item version (CESD-10). In terms of participant characteristics, none of the included studies reported whether women received antidepressant treatment. Regarding established GDM risk factors among participants, only limited information was available across studies; age, body mass index, family history of diabetes, preterm birth, marital status, and miscarriage were included in two trials (16, 17). The methodological quality assessment of included studies according to NOS is shown in **Tables 1**, **2**, and eight studies were high-quality.

**Table 1:** Characteristics of eligible studies.

Author	Year	Country	Design	Sample size
Tang	2020	China	Prospective cohort	1,426
Dong	2019	China	Prospective cohort	1,554
Huang	2016	China	Case-report	3,629
Dahlen	2015	Australia	Retrospective cohort	3,092
Versteegen	2021	USA	Prospective cohort	300
Hinkle	2016	USA	Prospective cohort	2,477
Morrison	2016	USA	Prospective cohort	1,021
Bowers	2013	USA	Retrospective cohort	111,952

**Table 2:** Characteristics of participants. .

Author	Year	Age	GDM diagnostic criteria	Depression measurement time	Evaluation scale	Covariates	NOS score
Tang	2020	28.6	OGTT	8-14 gestational weeks	SDS	Age, body mass index, family history of diabetes, education, smoking, drinking, income, parity, occupational	6
Dong	2019	29.8	OGTT	Before 20 weeks of pregnancy	EPDS	Age, body mass index, family history of diabetes, preterm birth	7
Huang	2016	27.1	–	Before 24 weeks of pregnancy	HAD	–	6
Dahlen	2015	29.0	–	–	EPDS	Age, body mass index, parity, smoking, race	7
Versteegen	2021	28.54	OGTT	–	EPDS	Age, body mass index	7
Hinkle	2016	28.13	OGTT	8-13 gestational weeks	EPDS	Age, body mass index, marital status, education, race	6
Morrison	2016	26.05	–	Early pregnancy stage	CESD-10	Age, body mass index, education, income, marital status	7
Bowers	2013	27.6	ICD-9	Prior to GD	ICD-9	age, race, parity, body mass index	8

OGTT, oral glucose tolerance test; GDM, gestational diabetes mellitus; SDS, Self-Rating Depression Scale; EPDS, Edinburgh Postnatal Depression Scale; HAD, hospital anxiety and depression; CESD-10, Center for Epidemiologic Studies Depression Scale-10 item version; ICD: International Classification of Diseases; NOS, Newcastle–Ottawa Scale.

### Synthesis of results

**Figure 2:**
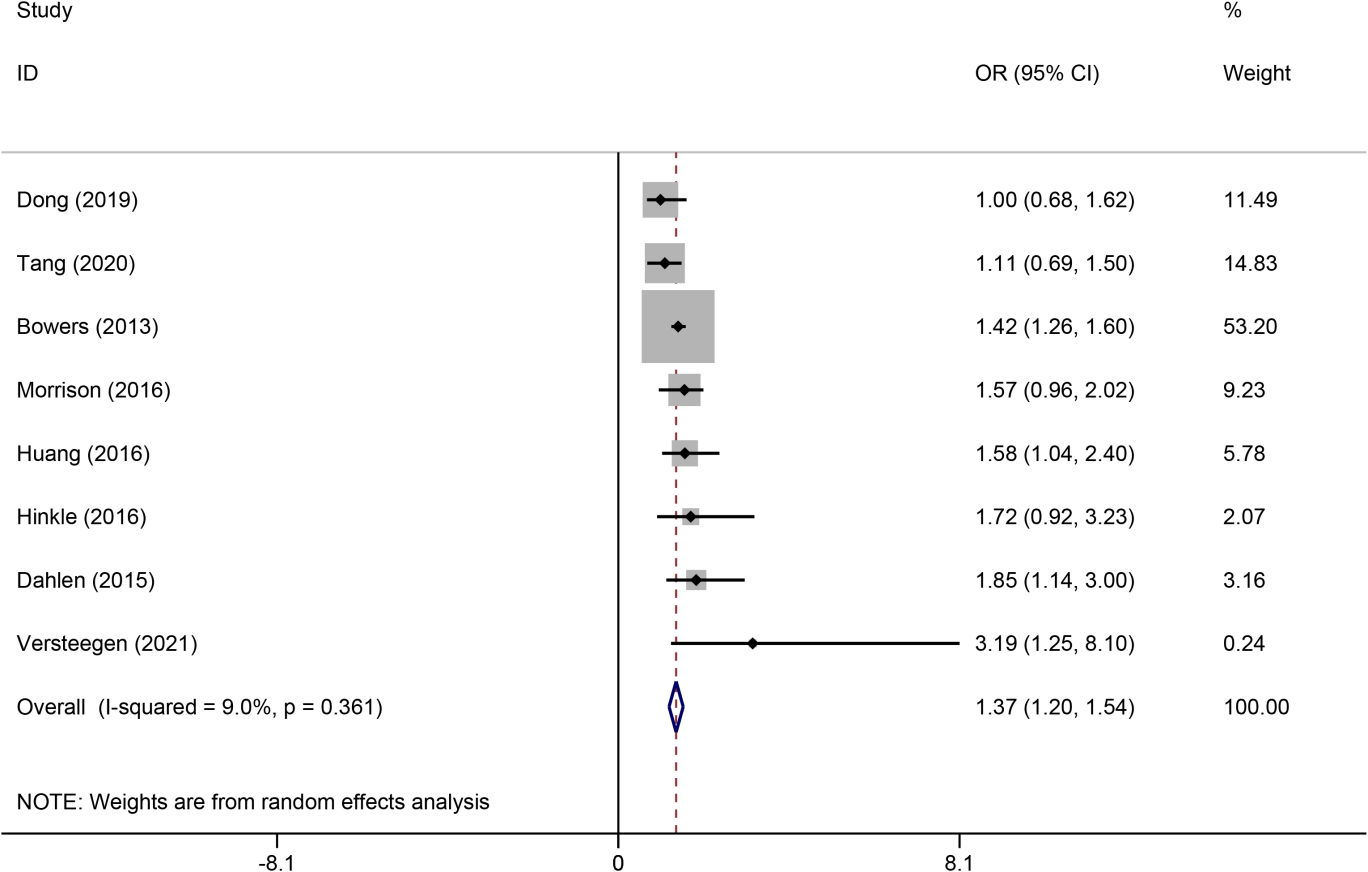
Forest plot of the effect of maternal depression on the risk of GDM in pregnant women.

The pooled analysis showed that maternal depression during pregnancy was significantly associated with an increased risk of GDM. The combined effect estimate was OR = 1.37, 95% CI: 1.20-1.54, p < 0.001 (**Figure 2**), with a low heterogeneity (I^2^ = 9.0%; P = 0.361). In addition, subgroup analyses were stratified by country, depression assessment tool, and study design; the pooled results remained consistent, with no significant differences observed across subgroups (**Table 3**).

**Table 3:** Subgroup analysis.

Subgroup	Group	No. of studies	Overall effect			Heterogeneity	
			OR (95% CI)	Z-score	p-value	*I^2^* (%)	*p*-value
Country						0	0.373
	China	3	1.15 (0.87, 1.43)	8.03	0.001	–	–
	Australia	1	1.85 (0.92, 2.78)	3.90	0.001	0	0.680
	USA	4	1.44 (1.28, 1.60)	17.67	0.001		
Design							
	Prospective cohort	5	1.25 (0.94, 1.55)	8.10	0.001	16.9	0.307
	Case report	1	1.58 (0.90, 2.26)	4.55	0.001	–	–
	Retrospective cohort	2	1.43 (1.27, 1.60)	16.81	0.001	0	0.373
Evaluation scale						–	–
	SDS	1	1.11 (0.70, 1.51)	5.32	0.001	–	–
	HAD	1	1.58 (0.90, 2.26)	4.55	0.001	34.1	0.208
	EPDS	4	1.44 (0.83, 2.04)	4.66	0.001	–	–
	CESD-10	1	1.57 (1.04, 2.10)	5.81	0.001	–	–
	ICD-9	1	1.42 (1.25, 1.59)	16.37	0.001	–	–

SDS, Self-Rating Depression Scale; EPDS, Edinburgh Postnatal Depression Scale; HAD, hospital anxiety and depression; CESD-10, Center for Epidemiologic Studies Depression Scale-10 item version; ICD: International Classification of Diseases.

### Sensitivity analysis

**Figure 3:**
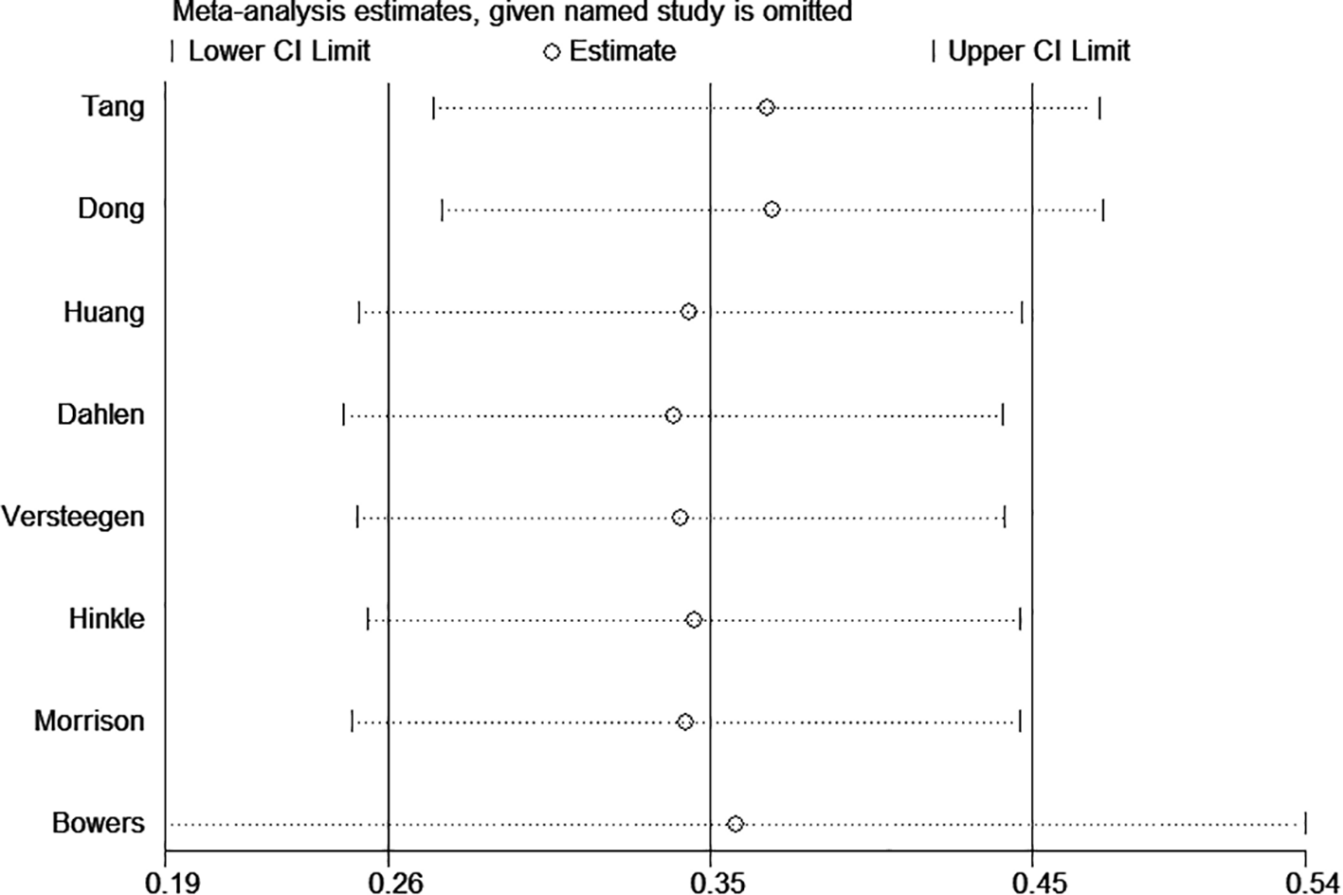
Sensitivity analysis of the GDM risk.

To assess the robustness of the pooled results, we performed a sensitivity analysis by sequentially excluding individual studies. Specifically, we excluded the study with the largest sample size (21) and the study with an excessively wide 95% confidence interval (18) (**Figure 3**). The exclusion of these studies did not materially alter the overall effect estimates, suggesting that the findings of this meta-analysis are stable and not driven by any single study.

### Publication bias

**Figure 4:**
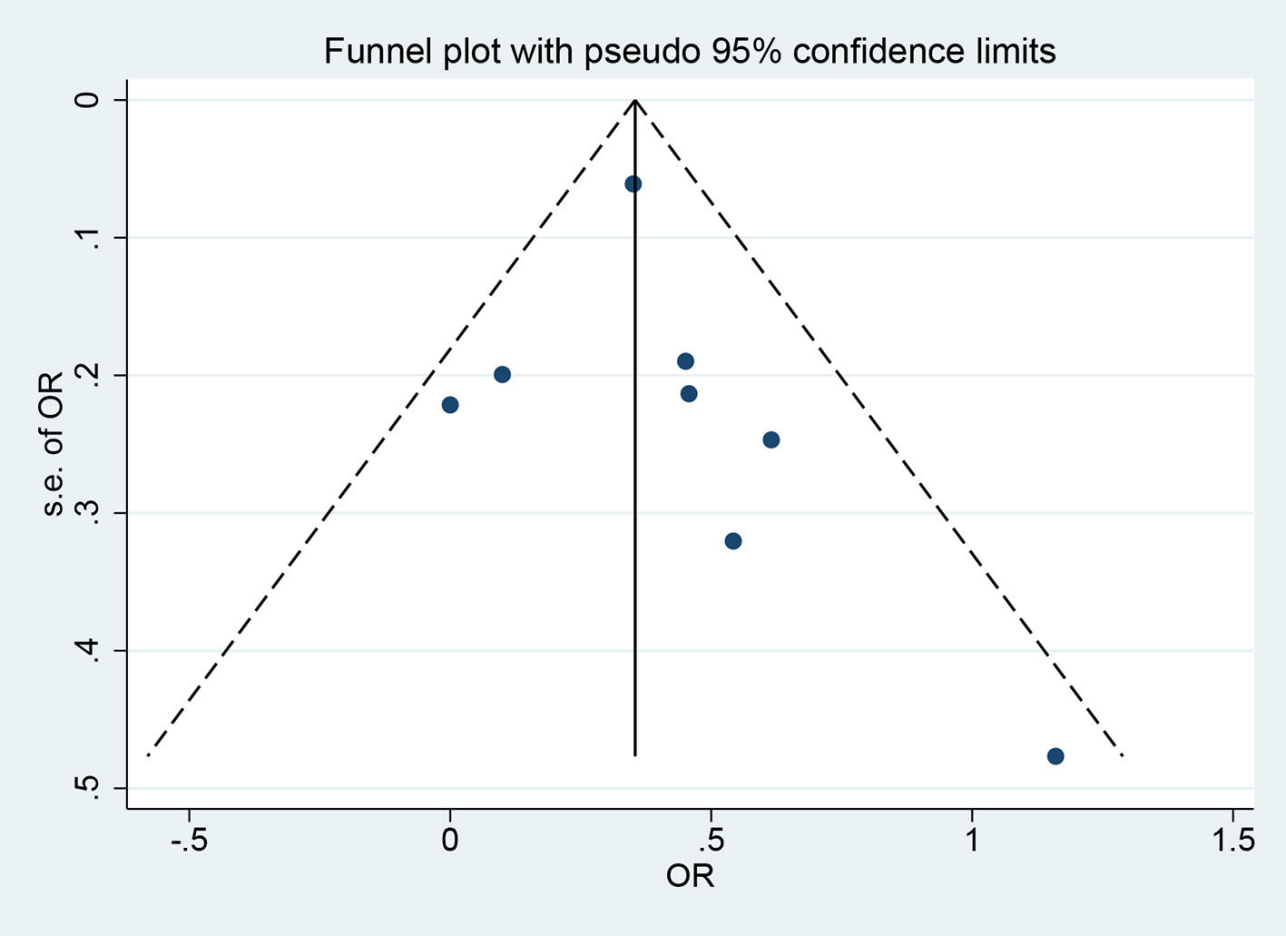
Funnel plot of the GDM risk.

The funnel plot and Egger’s test were utilized to evaluate publication bias. Visual inspection of the funnel plot showed a roughly symmetrical distribution of the included studies (**Figure 4**). The Egger’s test (P = 0.509) and Begg’s test (P = 0.138) indicated no significant publication bias.

## Discussion

In this meta-analysis, we found a significant association between maternal depression during pregnancy and an increased risk of GDM. Subgroup analyses based on country, depression assessment scale, and study design yielded consistent results, with no significant differences observed across subgroups. Sensitivity analyses, including the exclusion of the study with the largest sample size and the study with an excessively wide 95% confidence interval, did not affect the overall effect estimates. The pooled results across multiple studies indicated that women who experienced antenatal depression had a higher likelihood of developing GDM compared to those without depression (12, 14, 16). This finding was consistent across different geographic regions, and diagnostic methods for depression, suggesting a potentially independent relationship between maternal psychological status and glucose metabolism during pregnancy.

Our findings are in line with several recent cohort studies that reported a positive association between antenatal depression and increased GDM risk (12, 14). However, some studies failed to detect a significant link, possibly due to limited sample sizes or lack of adjustment for key confounding factors (15, 18). Notably, previous studies investigating GDM risk factors have primarily focused on biomedical or lifestyle determinants, often overlooking psychosocial variables such as antenatal depression. Our findings highlight the importance of including psychological factors in future research, which may provide a more comprehensive understanding of GDM etiology and support the development of integrative prevention strategies (22). Compared with the previous meta-analysis that included five studies (19), our study incorporated a total of eight studies. Among them, four studies were overlapping with those included in the previous analysis, while we also identified and included four additional studies that met our predefined inclusion criteria and were eligible. In addition, by integrating cohort studies from multiple databases, our research can more comprehensively assess the relationship between perinatal depression and the risk of GDM.

Several biological and behavioral mechanisms may underlie this association. Depression is known to activate the hypothalamic–pituitary–adrenal (HPA) axis, leading to elevated cortisol levels, which can induce insulin resistance and impair glucose tolerance (23, 24). In addition, depression is often accompanied by systemic inflammation and dysregulated immune responses, which have been implicated in the pathogenesis of GDM (25, 26). Lifestyle-related factors such as poor dietary habits, physical inactivity, reduced sleep quality, and non-adherence to prenatal care among women with depression may further contribute to metabolic disturbances during pregnancy (27, 28). Recent evidence suggests a potential association between antidepressant use during pregnancy and an increased risk of GDM (29, 30). A systematic review and meta-analysis conducted by Wang et al. revealed that exposure to antidepressant drugs during pregnancy significantly increases the risk of developing GDM (31). Since the studies we included did not provide data on antidepressant treatment, we were unable to assess its independent effect or conduct subgroup analysis. Further research is needed to clarify the relationship between antidepressant use during pregnancy and GDM, particularly regarding specific drug classes and their mechanisms of action. Healthcare providers should carefully consider the potential risks and benefits when prescribing antidepressants to pregnant individuals.

These findings highlight the importance of incorporating routine mental health screening into prenatal care, not only for psychological well-being but also for potential metabolic consequences. For example, routine screening for depressive symptoms in early pregnancy (for example with EPDS) could identify women at elevated metabolic risk; elevated antenatal depression scores might reasonably trigger earlier glucose evaluation (e.g., earlier OGTT), or more timely lifestyle guidance.

Several limitations should be acknowledged. First, most included studies were observational, and although adjusted for confounders, the possibility of residual confounding cannot be completely ruled out. Second, the included studies used different depression screening tools (e.g., EPDS, CESD-10, and HAD), which may introduce variability and subjectivity in exposure assessment. Third, GDM was diagnosed according to different criteria, which could contribute to outcome misclassification. Four, although statistical analyses did not suggest publication bias, the limited number of included studies means that bias cannot be entirely excluded. Finally, future research should prioritize prospective designs with standardized depression measures, unified GDM diagnostic criteria, and biomarker- or genetics-supported approaches to validate and further elucidate the antenatal depression–GDM link.

## Conclusion

This meta-analysis suggested that antenatal depression is associated with an increased risk of GDM. Screening for depressive symptoms early in pregnancy may improve the identification of women at higher GDM risk and enable timely intervention. In addition, integrating routine depression screening into prenatal care could provide clinicians with an opportunity for earlier glucose monitoring, personalized prevention strategies, and improved maternal–fetal outcomes.

## Data Availability

The original contributions presented in the study are included in the article/Supplementary Material. Further inquiries can be directed to the corresponding author.
